# Reply to Moretti et al. Would Moving Forward Mean Going Back? Comment on “Maselli et al. Direct Access to Physical Therapy: Should Italy Move Forward? *Int. J. Environ. Res. Public Health* 2022, *19*, 555”

**DOI:** 10.3390/ijerph19084620

**Published:** 2022-04-12

**Authors:** Filippo Maselli, Leonardo Piano, Simone Cecchetto, Lorenzo Storari, Giacomo Rossettini, Firas Mourad

**Affiliations:** 1 Department of Neurosciences, Rehabilitation, Ophthalmology, Genetic and Maternal Infantile Sciences (DINOGMI), Campus of Savona, University of Genova, 17100 Savona, Italy; 4313618@studenti.unige.it (F.M.); lorenzo.storari93@gmail.com (L.S.); 2 Sovrintendenza Sanitaria Regionale Puglia INAIL, 70126 Bari, Italy; 3 Fondazione dei Santi Lorenzo e Teobaldo, 12050 Rodello, Italy; l.piano@hotmail.it; 4 Direction of Health Professions, APSS, 38014 Trento, Italy; simone.cek@gmail.com; 5 School of Physiotherapy, University of Verona, 37129 Verona, Italy; giacomo.rossettini@gmail.com; 6 Department of Physiotherapy, LUNEX International University of Health, Exercise and Sports, 4671 Luxembourg, Luxembourg; 7 Luxembourg Health & Sport Sciences Research Institute A.s.b.l., Avenue du Parc des Sports 50, 4671 Luxembourg, Luxembourg

We want to thank you for the opportunity to respond to the issues raised in the letter to the Editor of Dr. Moretti et al. [1]. We would also like to thank Dr. Moretti et al. for their interest in our Communication [2] and for taking the time to express their concerns.

Physical therapy worldwide is increasing its profile, roles, and responsibilities. The World Physiotherapy (a subgroup of the World Health Organization) advocates direct access to physical therapy (DAPT) and patient/client self-referral [3]. In several countries, the physical therapy practice advances to a more independent care model, moving to the DAPT pathway [3]. We were expecting, and we are glad to have raised scholars’ interest in this topic. This scientific debate again underlines the relevance of DAPT, and the related challenges contextualized within each country, culture, professional education and regulation, healthcare system organization and history; also, it reflects the need for new emerging care models and professionals’ competencies to counteract the economic burden of health system expenses and satisfy the requirements of cost-effective solutions for the citizens who have musculoskeletal disorders (MSD).

In their letter to the Editor, Moretti et al. raised four main issues: (I) the quality of the included studies for DAPT effectiveness and safety; (II) the knowledge of physical therapists (PTs); (III) the Italian regulation; and (IV) the illustration within our communication. We will reply to each of their concerns below, thus allowing us to clarify our perspective further.

From their letter, it would appear that they believe that our Communication [2] is mainly based only on two studies, namely, Gagnon et al. (2020) [4] and Bishop et al. (2017) [5]. Notably, Moretti et al. raised concerns on a feasibility study that included almost one thousand patients [5]; that is, Bishop et al. [5], attempting to analyze all the relevant factors—including recruitment, economic, safety and clinical effectiveness—to inform and justify an ongoing full randomized controlled trial (RCT), ascertain that both clinical and cost outcomes were similar for the PT-led and general practitioner (GP)-led pathways. Furthermore, no safety issues were identified. In line with a more inclusive healthcare system and to ensure that patients’ demands can be met in primary care, findings from this pilot trial in United Kingdom (UK) further validated the need for a future larger RCT that will provide high-quality evidence about the clinical and cost-effectiveness of DAPT for patients with MSD. Accordingly, the results of Bishop et al. [5] were confirmed more recently by Downie et al. with a 2-year service evaluation of UK primary care data [6]. Moretti et al. also argue that the main findings from Gagnon et al. (2020) [4] had no clinical differences between physician-led (i.e., usual care) and PT-led management in the emergency department (ED) for the primary outcome measures (i.e., Brief Pain Inventory and Numeric Pain Rating Scale) [4]. In this study, “participants—with minor MSD (i.e., not requiring an emergency care pathway)—in the PT group were initially assessed by a PT following nurse triage”; the PT “Interventions were then recommended based on the clinical analysis and PT diagnosis, including advice, technical aids, imaging, prescribed or over-the-counter medication, and consults with other health care professionals”. Notably, the participants of this group only encounter the “ED physician prior to discharge”. Perhaps the reason for their concerns lies in the fact that our intention is not to determine which pathway is the most effective; instead, we would like scholars and institutions to focus on novel solutions, to exploit resources and competencies that are still little known and utilized, and to discuss the potential benefits for both the patient and the systems. In fact, there is no doubt that both physicians and PTs effectively treat their patients, supported by the best available evidence. However, in addition to statistical differences between interventions, we believe that the effectiveness and appropriateness of healthcare services must also be measured through several direct and indirect measures (i.e., hospitalization rate, medication prescription and intake, imaging usage, and secondary care referral), which were observed superior for the PT-led pathway in the Gagnon et al. study [4]. Recently, other recent European experiences reported the same advantages for both DAPT or direct triaging to physical therapy in primary care, to support clinical, political and economic solutions to the ongoing demands of citizens and systems [7,8,9,10].

However, we would also like to rebut that several recent reviews were cited in our Communication [2]. While we make no formal claims to provide a comprehensive or formal review on the DAPT, we believe it helps to look at the general trend of outcomes from recent systematic reviews on this topic. For example, since 2014, seven reviews have been conducted, investigating the comparative effectiveness and safety of PT-led and GP-led pathways [11,12,13,14,15,16,17]. All these confirmed the safety and positive effects regarding the clinical outcomes for both. A general lack of homogeneity in the available literature makes it difficult for a truly valid comparison among the studies. However, the cost-effectiveness of the PT-led pathway has been demonstrated. In addition, a broad range of studies reported that DAPT has some advantages, including medication intake reduction, lost workdays, number of imaging investigations, hospitalization rate, waiting time, referral appropriateness, economic burden and patients’ satisfaction [12,14,15,16,17]. The latter aspects should not be underestimated, especially after the strong impact and subsequent crisis of the health systems due to the COVID-19 pandemic [18,19,20,21,22,23,24]. The need to cope with hospital overload and medical doctor shortages clearly emerged; they were struggling to meet patient demand for appointments [25].

Secondly, Moretti et al. perfectly hit the nail on the head: Child et al. observed higher levels of knowledge in managing MSD than several physicians, except for orthopedic physicians, the medical specialty for MSD [26]. Accordingly, the highly specialized physician plays a key role when patients are referred for advanced management in the secondary care line. Similarly, based on Child et al. the utilization of PTs in primary care and as first contact professionals for minor and mild MSD seem to be suitable, with direct implications for both health and public policy decisions [26]. Accordingly, PTs have also been shown to possess the ability for higher selection accuracy for appropriate orthopedic consultation referral for those patients in need of more specific interventions [6,27]. Notably, PTs possessing a musculoskeletal specialization were observed to have higher knowledge for the management of MSD than licensed colleagues [26]. Moreover, according to “article 6 of Law 43 of the 1st of February 2006” [28], the musculoskeletal specialization in physical therapy is earned during a two years postgraduate program (namely, a Master degree), which follow international educational standards [29]. Furthermore, these programs (actually, six in Italy [30,31,32,33,34]) are constantly monitored by the International Federation of Orthopedic Manipulative Physical Therapists—a World Physiotherapy full member [35]—which make these competencies generalizable in the Italian territory also. All the above provide a solid guarantee of the potential role of physical therapy within primary care, being the perfect interlocutor for orthopedic physicians in secondary care.

Third, Moretti et al. questioned the autonomy and the diagnosis of physical therapists in Italy. The term derives from the Latin diagnōsis, through the ancient Greek διάγνωσις (diágnōsis), from διαγιγνώσκειν (diaghignóskein, to understand), formed by διά (diá, through) + γιγνώσκειν (ghignóskein, to know). In broad terms, diagnosis is thus the identification of the nature and/or cause of something, of whatever nature. From this point of view, the term functional diagnosis and functional evaluation do not differ. For further clarity, in our Communication [2], we refer to the functional diagnosis/evaluation based on the broadly accepted International Classification of Functioning [36]. Instead, medical diagnosis effectively ascribes to a causal claim of a different nature and suggests a biomedical explanation of illness. From this perspective, a diagnosis also possesses legal and political implications, providing the citizens the opportunity to access welfare benefits. It also plays a significant social function by validating illness [37]. In other words, the main differences between medical and physical therapy diagnoses are as follows: the former focuses on the causes of a certain disease/injury, while the latter mainly focuses on the consequence of such disease/injury [38]. Medical diagnosis converges on the pathoanatomical cause of a disease; instead, physical therapy diagnosis focus on the limitation in performing activities and restriction in participation [38]. Notably, Article 1 of the Ministry of Health Decree No. 741 of 14 September 1994, ‘Regulations concerning the identification of the position and related professional profile of the physical therapist’, reported by Moretti et al. states that “The physical therapist profession is identified with the following profile: the physical therapist is the healthcare professional, … who provide prevention, care and rehabilitative interventions, autonomously, or in collaboration with other healthcare professionals, within the areas of motor, upper cortical and visceral functions resulting from pathological events of various etiologies, congenital or acquired” [39]. We would also like to add a few considerations on the following statement, “the involvement of physical therapists in Italy … is always secondary to both diagnosis and prescriptions performed by the physician, also in private practice”. In the public health system, the majority of outpatient specialist services/pathways are secondary to both the diagnosis and prescriptions performed by the physician. However, it should also be said that there exists areas in which the physical therapists practice autonomously as part of a multidisciplinary team even in the temporary absence of a diagnosis (e.g., developmental disabilities) or situations in which physical therapists provide consultancy (e.g., acute care units), which may result in direct physical therapy intervention [40]. Moreover, in private practice, the DAPT are based on the free choice of the citizen, who has the full right to decide which pathway of care, established via informed consent, to rely on. Then, the citizen can self-refer to the physical therapist, which has the professional responsibility to triage whether the patient’s condition is within the scope of practice or whether the patient requires a referral to another heath professional [41]. According to Article 40 of the Code of Ethics, physical therapists know that it is always appropriate to invite the patients to keep their general practitioner informed and may also send a report following the person’s consent or request [42]; that is, the concept of physical therapists’ subalternity and sector-health are longer plausible or realistic. In fact, Law 3/2018 [43] established an ordinal level for health professionals, and Law 24/2017 [44] modified the penal responsibility that falls on the individual professional who does not operate according to the best practices and guidelines. These regulatory changes treat all health professions on an equal level.

Finally, we would like to raise our concerns about their comments on Figure 1 [2]. We found it unfair to support the concept of a “one-man show” approach only based on an illustration. We further clarified and supported the benefits that DAPT can provide at all levels of healthcare, primarily to patients. We also illustrated the medical professions’ advantages, especially in terms of the management appropriateness recommended by clinical practice guidelines for managing several MSDs [45,46,47,48,49], including the ones reported by Moretti et al. [50,51,52,53]. To deny this is antithetical given the global trend, the currently available evidence, the concept of multidisciplinary rehabilitation and the patient-centered model of care [54,55]. Furthermore, the citizen has the right to choose which professionals to self-refer for their health; to argue the opposite could represent a paternalistic approach to medicine, in contrast to the biopsychosocial model advocated by Moretti et al. [56]. Instead, we would affirm that DAPT is one of the strategies to be implemented to meet the ten areas of intervention recommended by the WHO Rehabilitation 2030 call to action, especially areas three (“Improving integration of rehabilitation into the health sector and strengthening inter-sectoral links to effectively and efficiently meet population needs”) and six (“Developing a strong multidisciplinary rehabilitation workforce that is suitable for country context, and promoting rehabilitation concepts across all health workforce education”) [57]. Other countries have already experienced that the positive overlap of competencies between every protagonist of rehabilitation leads to a better quality of the health services with the final goal to provide the best management option at the lowest cost for the citizens [58].

In summary, our goal was to provide our perspective and the current evidence on the DAPT; we also wanted to highlight a topic that we felt too little debated, but which has the potential to benefit the system if all players focus on a fairer and more citizen-centered healthcare system. According to Moretti et al. we firmly believe cooperation is a mainstay of modern rehabilitative medicine. However, we would still affirm our advocacy for recognizing the PT as the appropriate case manager for MSD, which should represent an opportunity for improving the quality of care rather than being a corporative issue. Any change in the practice and the norms of science must start from revisioning the underlying concepts motivating such practices and norms (i.e., ontology). A foundational change in ontology ought to lead to a change in norms and practices, and it should also challenge the way medicine and healthcare are organized, managed and financed. We call then for a genuine ontological shift, which must start from focusing on the benefits for singular citizens, instead of the dualism between isolated professional ‘silos’ [56,59].
